# Identification of Quality-Related Genomic Regions and Candidate Genes in Silage Maize by Combining GWAS and Meta-Analysis

**DOI:** 10.3390/plants14152250

**Published:** 2025-07-22

**Authors:** Yantian Lu, Yongfu Ding, Can Xu, Shubin Chen, Chunlan Xia, Li Zhang, Zhiqing Sang, Zhanqin Zhang

**Affiliations:** Xinjiang Academy of Agricultural and Reclamation Sciences, Shihezi 832000, China; lyt204712@163.com (Y.L.); 18893810043@163.com (Y.D.); 18299076107@163.com (C.X.); btcorn@163.com (S.C.); 17709939081@163.com (C.X.); 18149931215@163.com (L.Z.)

**Keywords:** silage maize, GWAS, meta-analysis, consensus quantitative trait loci, stalk quality

## Abstract

Enhancing quality traits is a primary objective in silage maize breeding programs. The use of genome-wide association studies (GWAS) for quality traits, in combination with the integration of genetic resources, presents an opportunity to identify crucial genomic regions and candidate genes influencing silage maize quality. In this study, a GWAS was conducted on 580 inbred lines of silage maize, and a meta-analysis was performed on 477 quantitative trait loci (QTLs) from 34 studies. The analysis identified 27 significant single nucleotide polymorphisms (SNPs) and 87 consensus QTLs (cQTLs), with 7 cQTLs associated with multiple quality traits. By integrating the SNPs identified through association mapping, one SNP was found to overlap with the cQTL interval related to crude protein, neutral detergent fiber, and starch content. Furthermore, enrichment analysis predicted 300 and 5669 candidate genes through GWAS and meta-analysis, respectively, highlighting pathways such as cellular metabolism, the biosynthesis of secondary metabolites, ribosome function, carbon metabolism, protein processing in the endoplasmic reticulum, and amino acid biosynthesis. The examination of 13 candidate genes from three co-located regions revealed *Zm00001d050977* as a cytochrome P450 family gene, while the other 2 genes primarily encode proteins involved in stress responses and other biological pathways. In conclusion, this research presents a methodology combining GWAS and meta-analysis to identify genomic regions and potential genes influencing quality traits in silage maize. These findings serve as a foundation for the identification of significant QTLs and candidate genes crucial for improving silage maize quality.

## 1. Introduction

Maize (*Zea mays* L.) is a significant crop utilized for both human consumption and animal feed. Globally, over 70% of maize is processed for livestock feed, particularly silage maize, which is known for its high nutritional content and energy density, making it a crucial feed for ruminant animals [1,2]. Enhancing silage maize quality traits poses a challenge in conventional breeding due to the considerable amount of time and cost involved in improving grain and straw quality traits. The advent of molecular marker systems enables the identification of genomic regions with substantial impacts on silage maize quality. Leveraging these regions through molecular breeding offers a promising approach to addressing this challenge [3,4]. The initial documentation of QTL mapping for silage maize quality traits dates back to 1997 [5,6]. Subsequent studies have identified QTLs associated with diverse silage traits. To expedite advancements in silage quality through molecular breeding techniques, precise mapping, cloning, and integration of advantageous genetic loci or genes from diverse germplasm resources are imperative.

With the rapid development of genotyping technologies and the application of quantitative genetic methods, hundreds of QTLs related to silage quality have been located since 1980, yet the number of cloned genes remains relatively small. QTLs and markers related to silage quality are essential for the effective application of molecular marker-assisted breeding. However, due to the influence of genetic background, population size and type, and statistical methods, QTLs identified in parental mapping populations have often not been suitable for marker-assisted selection or gene cloning. Therefore, it is necessary to identify stable QTLs that have a significant effect on quality traits [7]. Genome-wide association analysis (GWAS), an alternative approach to QTL mapping, has shown that loci associated with traits identified through GWAS are also confirmed in parental QTL mapping. This highlights the advantages of integrating both methodologies to pinpoint regions linked to specific traits [8]. The genome-wide association study (GWAS) of 368 inbred lines across seven environments revealed associations between 73 single nucleotide polymorphisms (SNPs) and acid detergent fiber (ADF), as well as 41 SNPs and neutral detergent fiber (NDF). The identification of the *ZmC3H2* gene, which encodes *p*-coumarin hydroxylase, as closely linked to the biosynthesis of ADF and NDF, is noteworthy [9]. Linkage analysis has been instrumental in identifying numerous QTLs associated with silage quality and cell wall degradation. Meta-QTL (MQTL) analysis has further enabled the examination of QTLs related to quality and digestion. Notably, 42% of MQTLs were linked to quality traits, 356 QTLs were associated with cell wall synthesis, and 39% of candidate genes were situated within the confidence interval of MQTLs [10,11].

Numerous QTLs and SNPs have been documented in association with silage quality traits. Meta-analysis employs mathematical models to enhance the precision of QTL confidence intervals by amalgamating diverse QTL mapping studies, thereby improving the accuracy and efficacy of QTL identification [12,13]. Meta-analysis has been employed in QTL integration, consensus map construction, and the fine mapping of candidate genes for important maize traits [14,15].

This study aims to enhance the integration of GWAS and MQTL in order to pinpoint genomic regions and key candidate genes influencing the inheritance of silage maize quality. This approach improves the understanding of the genetic basis underlying silage quality, thereby facilitating the discovery, cloning, and application of pivotal QTLs or candidate genes in silage maize breeding.

## 2. Materials and Methods

### 2.1. Experimental Materials and Design

Seven sets of germplasm, comprising 580 Asia-adapted breeding lines from early maturing improvement groups from Canada, BSSS (Iowa Stiff Stalk Synthetic) lines, NSS (Non-Stiff Stalk) lines, Huanghuaihai germplasm, the European KWS series, and the Pioneer series, were used to generate the experimental datasets for this study. These lines were planted in the maize breeding experimental field of the Crop Research Institute of the Xinjiang Academy of Agricultural Reclamation Sciences in 2020 (44.31° N, 85.99° E). The planting density was 105,000 plants/ha. The field experiment followed a randomized block design with two replications. In each replication, each entry was planted in a single 4.5 m row with a row spacing of 0.55 m. Field management was consistent with standard production practices.

This study utilized phenotypic data obtained from a single-year trial at one location. Although multi-environment trials would further confirm the stability of trait associations, the substantial sample size and extensive genetic diversity among the 580 maize inbred lines significantly strengthened the reliability and applicability of the identified genomic associations. Future studies may extend these findings by incorporating multi-year and multi-location trials.

### 2.2. Phenotypic Data Acquisition and Processing

At the 3/4 milk stage of maize development, five adjacent plants were harvested from each plot, excluding edge plants. The entire plant material was crushed and dried until a constant weight was achieved. The contents of crude protein (CP), crude fat (CF), starch, acid detergent fiber (ADF), and neutral detergent fiber (NDF) were analyzed using a FOSS near-infrared analyzer (NIRS DS250) as the reference method for subsequent quality trait assessment. Each sample was measured in triplicate. Preliminary data summarization, mean calculation, and basic descriptive statistics for each trait were performed using Excel 2019. R 4.2.2 software was then used for detailed statistical analyses, including correlation and principal component analysis (PCA).

The PCA was conducted using phenotypic trait data to visualize the clustering of maize inbred lines based on trait similarity. The PCA results were visualized as a scatterplot to highlight sample differentiation rather than trait grouping. Pairwise correlations among traits were analyzed separately to clearly identify trait-to-trait relationships.

### 2.3. Genome-Wide Association Analysis

The SNP genotyping data were obtained for each sample using the Maize SNP 40K Genotyping by Targeted Sequencing (GBTS) method. Genotype data quality control was conducted using PLINK 1.9 software with the following screening criteria: minimum allele frequency (MAF) > 0.05 and missing rate < 0.05. Additionally, linkage disequilibrium (LD) quality control was performed using a window size of 100 kb, step size of 1, and *r*^2^ = 0.05, resulting in the identification of 40,124 high-quality SNP markers. Whole-genome association analysis was conducted using the FarmCPU model in GAPIT, while population structure was assessed using Structure 2.3.4 software. The optimal number of subgroups was determined based on the maximum likelihood function (LnP(D)) from the Structure 2.3.4 output and the ΔK method. An integration of results from multiple runs was performed using CLUMPP 2.0 software and visualized using R 4.1.3. PCA was conducted, and kinship and LD attenuation were calculated using TASSEL 5.0.

### 2.4. Collection and Collation of Meta-Analysis Data

QTL and mapping data related to CP, CF, starch, NDF, and ADF content in silage maize were compiled from the relevant literature accessed through the National Center for Biotechnology Information (NCBI), China Knowledge Network (CNKI), and Web of Science databases. The collected map data included details such as population type, size, chromosome, linkage group, marker designation, and location. QTL information comprised unique identifiers, chromosome and linkage group assignments, logarithm of the odds (LOD) scores, putative R^2^ values, QTL positions, and the start and end points of the QTL regions. In cases where the confidence interval (CI) was not reported in the QTL data, a 95% CI was estimated using the method described by Darvasi and Soller:

C.I. = 530/(N × R^2^) (1)

C.I. = 163/(N × R^2^) (2)

where C.I. refers to the 95% confidence interval of the QTL, N refers to the size of the mapping population, and R^2^ refers to the variance ratio explained by the QTL, that is, the contribution rate. Formula (1) was applied to the F_2_ and backcross populations, and Formula (2) was applied to the recombinant inbred line (RIL), doubled haploid (DH), and near isogenic line (NIL) populations.

### 2.5. Meta-Analysis of Quality-Related Traits

In this study, the term MQTL refers specifically to “Meta-QTL,” indicating QTL regions derived through meta-analysis by integrating multiple previously reported QTLs from independent studies. Original QTLs obtained directly from individual studies are referred to as QTLs. The term “cQTL” stands for “consensus QTL”, representing genomic regions identified through meta-analysis that integrate multiple previously reported QTLs into a refined and precise location. This meta-analytical approach enhances the precision and reliability of identified genomic regions by synthesizing and refining results from diverse genetic mapping studies.

The IBM 2008 Neighbors genetic map was used as the reference, and BioMercator 4.2 software was employed to compute the distance between shared markers using the sequence function. Molecular markers indicating the original QTL positions and confidence interval boundaries were plotted onto the reference map to establish a coherent framework. The mapping function within BioMercator 4.2 was then used to identify multi-QTL clusters.

### 2.6. Candidate Gene Mining of Quality-Related Traits in Silage Maize

To expand the gene mapping approach, the distribution range of the MQTLs was compared with significant loci associated with relevant traits identified by GWAS, in order to determine more reliable genomic regions. Candidate genes were analyzed through Gene Ontology (GO) and Kyoto Encyclopedia of Genes and Genomes (KEGG) enrichment analysis (https://www.omicshare.com/ (accessed on 1 February 2024)). The resulting data were integrated with information from the Maize Genetics and Genomics Database (https://www.maizegdb.org/ (accessed on 4 February 2024)) and the National Center for Biotechnology Information (https://www.ncbi.nlm.nih.gov/ (accessed on 4 February 2024)) to annotate and predict candidate genes.

## 3. Results

### 3.1. Phenotypic Analysis of Quality-Related Traits

**Figure 1:**
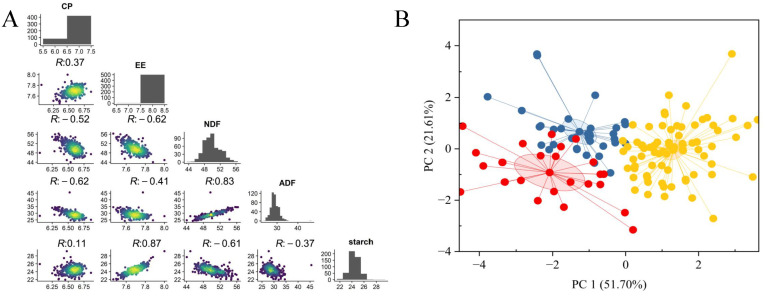
Phenotypic variation and relationships among silage maize quality traits. (**A**) Pairwise correlations among traits (CP, CF, NDF, ADF, starch), with correlation coefficients shown in the scatter plots. All correlations are statistically significant at *p* < 0.05. (**B**) PCA plot of maize inbred lines based on phenotypic data. Each dot represents one maize line. PC1 and PC2 explain 51.70% and 21.61% of total phenotypic variance, respectively, jointly accounting for 73.31% of the variation. Colors represent groupings based on phenotypic similarity identified by PCA.

The quality traits of silage maize exhibited substantial genetic variation, with coefficients of variation ranging from 1.20% to 7.14%. The skewness and kurtosis values indicated acceptable distributions for quantitative genetic analysis, although some kurtosis values slightly exceeded the ±1.0 range (Table 1). These values were typical of quantitative traits and thus suitable for subsequent GWAS analysis. Significant positive correlations were observed among the traits as follows: CP, CF, and starch; NDF and ADF; and CF and starch (Figure 1A). The PCA based on phenotypic data separated the maize inbred lines into distinct clusters (Figure 1B). The first two principal components explained 51.70% (PC1) and 21.61% (PC2) of the total variance, together accounting for 73.31% of the overall variation. The PCA highlighted clear differentiation among the maize inbred lines based on phenotypic profiles. However, PCA does not directly group traits; therefore, pairwise correlation analyses were conducted separately to investigate trait-to-trait relationships (Figure 1A). These results indicate that the five quality-related traits in silage maize inbred lines were interrelated, and their combined variation contributed to the observed differences among lines.

### 3.2. Analysis of Population Genetic Diversity and Genetic Relationships

**Figure 2:**
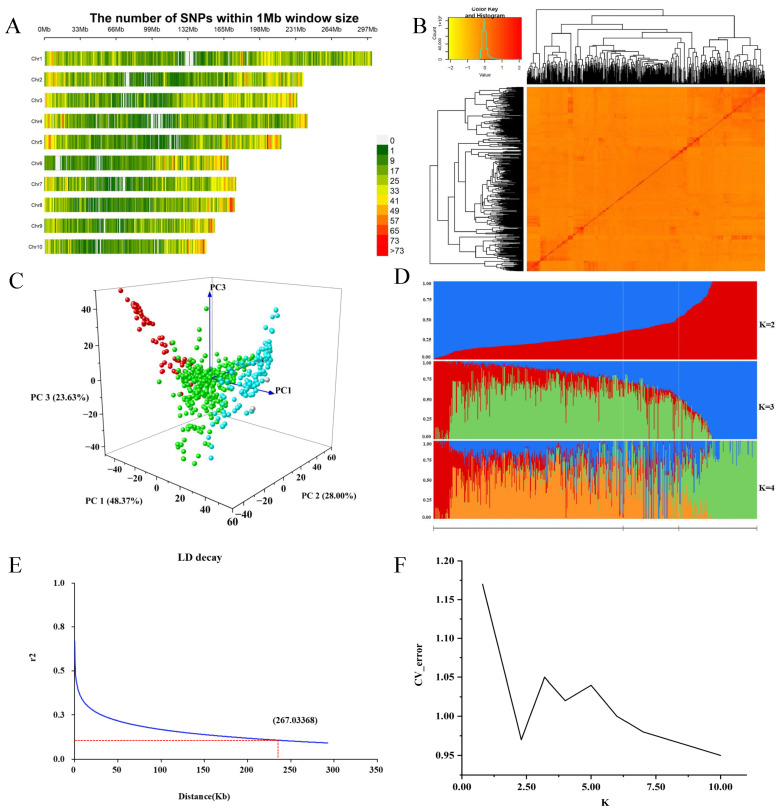
Analysis of population genetic diversity and genetic relationships. (**A**) Single nucleotide polymorphism (SNP) density distribution across chromosomes; (**B**) kinship heat map; (**C**) PCA showing genetic differentiation among 580 maize inbred lines (colors reflect clustering along PC1–PC3); (**D**) population structure of 580 inbred lines based on STRUCTURE analysis; (**E**) linkage disequilibrium decay; (**F**) ∆K value of 580 inbred lines based on 40,124 SNPs.

After quality control, 40,124 high-quality SNPs were mapped across all 10 chromosomes (Figure 2A). The number of SNPs per chromosome ranged from 2657 on chromosome 10 to 5641 on chromosome 1. The SNP distribution density in 1 Mb windows showed a tendency toward higher density at chromosome ends and lower density in the middle, although this pattern was not uniform across all chromosomes (Figure 2A).

Population structure analysis based on the 40,124 SNPs indicated that the 580 maize inbred lines could be grouped into two main subpopulations at K = 2, consisting of 141 and 439 lines, respectively. This grouping was supported by the cross-validation (CV) error curve (Figure 2F), which showed the lowest value at K = 2, suggesting this as the optimal structure for the panel. Additional analyses at K = 3 and K = 4 were included to illustrate finer-scale substructure, although increased admixture and reduced clarity were observed with higher K values (Figure 2D).

The PCA further confirmed broad genetic differentiation among the lines (Figure 2C), consistent with the major groupings identified by STRUCTURE at K = 2. This was further supported by the CV error curve (Figure 2F) and the linkage disequilibrium (LD) decay pattern, which provided additional insights into genome-wide resolution for association analysis (Figure 2E). The observed genetic clustering likely reflects differences in breeding history or geographic origin.

The kinship heat map revealed that most genetic relationship coefficients among the 580 maize inbred lines ranged between 0.3 and 0.5, indicating a relatively simple population structure (Figure 2B). This suggests that most inbred lines were either unrelated or only loosely related.

### 3.3. Genome-Wide Association Analysis of Quality-Related Traits

**Figure 3:**
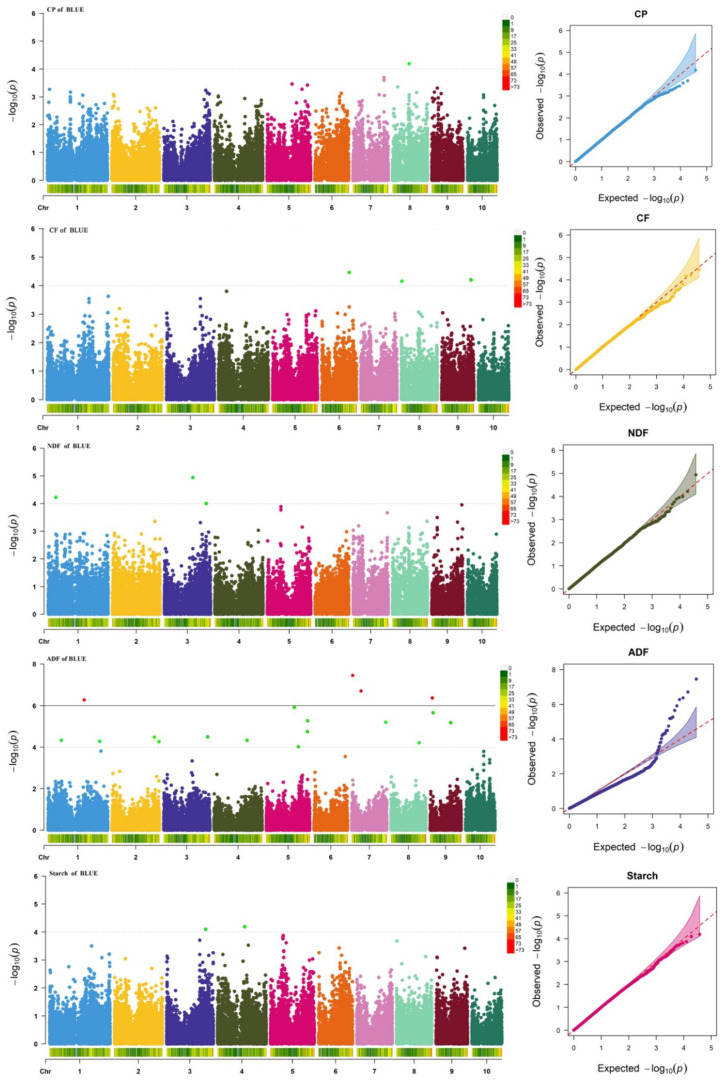
Manhattan and QQ plots for quality traits. CP: crude protein; CF: crude fat; NDF: neutral detergent fiber; ADF: acid detergent fiber; BLUE: best linear unbiased estimate. The dashed line indicates the significance threshold.

Using 40,124 high-quality SNPs and phenotypic data for silage maize quality traits, GWAS was performed using the FarmCPU model (Figure 3). PCA and kinship matrices were included as covariates to reduce potential confounding due to population structure and relatedness. The Manhattan and QQ plots demonstrated effective control of population stratification and spurious associations across traits (Figure 3). A total of 27 SNPs were significantly associated with the five quality-related traits (Supplementary Table S1). Specifically, 18 SNPs were associated with ADF, 3 with CF, 3 with NDF, 2 with starch, and 1 with CP. These significant loci were distributed as follows: ADF-related SNPs were located on chromosomes 1, 2, 3, 4, 5, 7, 8, and 9; CF-related SNPs on chromosomes 3, 4, and 6; NDF-related SNPs on chromosomes 4, 6, and 9; starch-related SNPs on chromosomes 8 and 9; and the CP-associated SNP was located on chromosome 1.

### 3.4. QTL Analysis of Quality-Related Traits in Silage Maize

**Figure 4:**
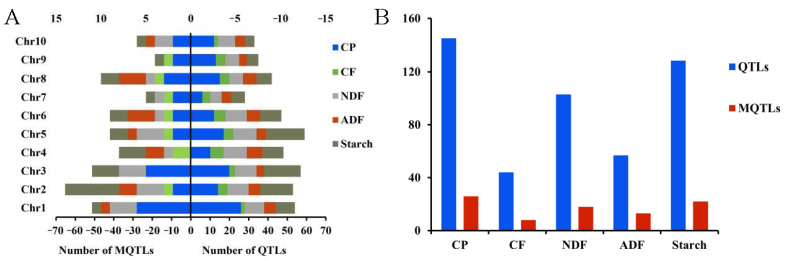
Distribution of quantitative trait loci (QTLs) and Meta-QTLs for quality-related traits. (**A**) Mirrored bar plot showing the chromosome-wise distribution of QTLs and MQTLs. Original QTLs (identified from previously published studies) are displayed on the right-hand side (positive lower *x*-axis), while MQTLs (Meta-QTLs identified by integrating multiple QTLs through meta-analysis) are shown on the left-hand side (mirrored on the negative upper *x*-axis). The use of negative values for MQTLs is solely for visual symmetry and does not indicate actual negative quantities. (**B**) Total number of QTLs and MQTLs identified for each trait. This comparison highlights the refinement achieved through meta-analysis across different quality traits (CP, CF, NDF, ADF, and starch).

Figure 4A,B show the distribution of previously published QTLs (individual QTLs collected from the literature) and MQTLs (Meta-QTLs), which represent consensus QTL regions identified through meta-analysis in this study, reflecting the integration of multiple QTL studies into consensus regions. A total of 456 quality-related QTLs were compiled from 59 mapping populations across 18 studies (Supplementary Table S2). The number of QTLs per trait and their distribution across the 10 maize chromosomes were uneven (Figure 4A). The highest number of QTLs (54) was located on chromosome 1, while the fewest (28) were on chromosome 7. Trait-wise, CP and starch had the most QTLs (145 and 114, respectively), while CF and ADF had the fewest (44 and 57, respectively) (Figure 4B). The number of QTLs per chromosome for each trait ranged from 2 to 20.

### 3.5. Construction of a “Consistent” Map for Quality Traits and QTL Meta-Analysis

**Figure 5:**
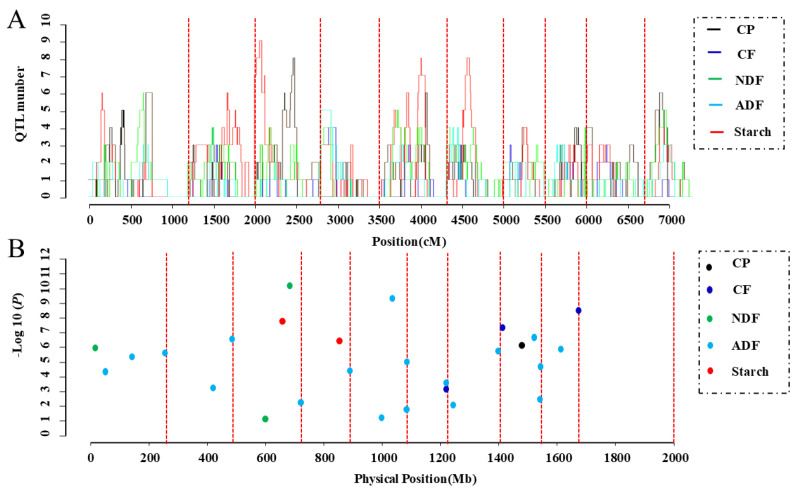
Significant single nucleotide polymorphisms (SNPs) were detected using quantitative trait locus (QTL) meta-analysis and association mapping (AM). (**A**) QTL meta-analysis based on previous reports, with each consensus QTL (cQTL) representing more than three original QTLs. (**B**) Significant SNPs located in this study. The 10 chromosomes are divided by red dotted lines and are arranged sequentially from chromosome 1 (leftmost) to chromosome 10 (rightmost). SNPs are plotted based on their physical positions and are correctly assigned to their respective chromosomes.

Using BioMercator 4.2 software and the IBM2 2008 Neighbors reference map, 456 QTLs related to silage quality were projected onto a unified “consistent” map (7300.9 cm), with an average marker interval of 29.47 cm. Meta-analysis identified 26, 8, 18, 13, and 22 consensus QTLs (cQTLs) for CP, CF, NDF, ADF, and starch, respectively. The physical length of individual QTL intervals ranged from 2.38 to 86.21 Mb (Supplementary Table S3). The cQTLs were unevenly distributed across maize chromosomes. A strong positive correlation was observed between QTL density and the number of cQTLs per chromosome (*R* = 0.90, *p* < 0.01). Traits with a higher number of QTLs also had more cQTLs. On chromosomes 2 and 4, cQTLs for starch overlapped with cQTLs for ADF and NDF, respectively. Additionally, three cQTLs related to ADF, NDF, and starch were located on chromosome 6. On chromosomes 7 and 10, CP-related cQTLs overlapped with starch-related cQTLs, and a starch-related cQTL overlapped with an ADF-related cQTL on chromosome 8 (Figure 5A,B).

A total of 5669 non-redundant genes were located within the cQTL regions across the five traits, including 1718, 630, 1213, 1016, and 1092 genes for CP, CF, NDF, ADF, and starch, respectively. Some of these genes have been cloned and functionally validated for quality-related traits.

### 3.6. Identification of Important Loci Using GWAS and Meta-Analysis

In this study, AM signals refer specifically to significant SNP associations identified through our GWAS using the FarmCPU model on 580 inbred lines. The term “AM signal” is used to distinguish these GWAS-derived results from cQTLs derived via meta-analysis. The mapping resolution of AM depends on the rate of LD decay. Due to the rapid LD decay in maize, AM can pinpoint QTLs at the gene level with high resolution. To identify key candidate loci for quality-related traits, the physical positions of cQTLs were compared with the positions of significant SNPs. Some significant SNPs overlapped with cQTL regions. Notably, AM signals corresponding to cQTLs were detected for CP, NDF, and starch, specifically within the intervals of qCP8-2, qNDF1-1, and qST4-3, respectively (Figure 3; Supplementary Table S3). This overlap between AM signals and cQTLs provides a strategy to prioritize stable loci for candidate gene identification. However, not all cQTLs corresponded to AM signals, which may reflect differences in detection power or population-specific effects between bi-parental QTL mapping and GWAS panels. Therefore, cQTLs without matching AM signals should be interpreted with caution.

### 3.7. Identification and Functional Analysis of Candidate Genes

Based on the significant SNPs identified in this population and considering linkage disequilibrium (LD), candidate genes related to silage quality traits were identified using the *B73_RefGen_v4* reference genome. A region of 267 kb upstream and downstream of each SNP was defined as the candidate interval. Within these intervals, a total of 300 potential candidate genes were identified for the five quality-related traits. In addition, 5669 non-redundant candidate genes were identified within the cQTL regions. Furthermore, one, six, and six possible candidate genes related to CP, NDF, and starch, respectively, were identified within three overlapping significant mapping intervals.

**Figure 6:**
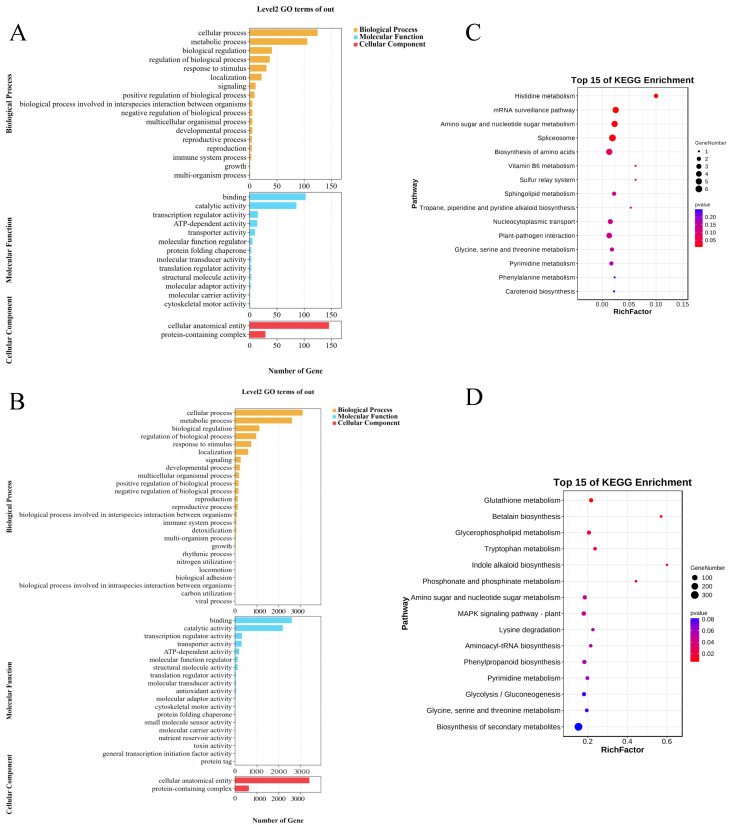
Candidate gene enrichment analysis (**A**) Gene Ontology (GO) enrichment analysis of genome-wide association analysis (GWAS) candidate genes. (**B**) GO enrichment analysis of meta-analysis candidate genes. (**C**) Kyoto Encyclopedia of Genes and Genomes (KEGG) enrichment analysis of GWAS candidate genes. (**D**) KEGG enrichment analysis of meta-analysis candidate genes.

The GO enrichment analysis of these candidate genes revealed that biological processes were mainly enriched in cellular processes, metabolic processes, biological regulation, and stimulus-response signal regulation. The main enriched cellular components included cellular structures and protein complexes, while the dominant molecular functions involved binding, catalytic activity, transcriptional regulatory activity, ATP-dependent activity, and transport activity (Figure 6A,B). KEGG pathway analysis indicated that the enriched pathways included metabolism, the biosynthesis of secondary metabolites, ribosome function, carbon metabolism, protein processing in the endoplasmic reticulum, and amino acid biosynthesis (Figure 6C,D).

To identify key genes associated with silage maize quality, the candidate genes were further screened based on their GO terms and shared KEGG pathway annotations. Thirteen candidate genes were annotated, mainly encoding proteins and transcripts involved in biosynthetic pathways and ribosomal functions; however, no candidate gene directly related to CP was identified (Supplementary Table S4).

It is important to note that the candidate genes identified in this study represent predictive associations based on genomic location and existing functional annotations. While these genes are promising for further investigation, functional validation through transcriptomic approaches (e.g., RNA-seq) or molecular assays (e.g., RT-qPCR) is necessary to confirm their roles in regulating silage maize quality traits. Such validation is beyond the scope of the current study; therefore, the reported genes serve as foundational targets for future experimental research aimed at advancing silage maize breeding programs.

## 4. Discussions

### 4.1. Phenotypic Structure Variation in the Associated Population

In this study, phenotypic data were collected from a single location during one growing season, which limits direct inference regarding genotype-by-environment interactions. However, this limitation is substantially mitigated by the large sample size and the high genetic diversity among the maize inbred lines used, increasing confidence in the robustness of the reported genomic associations. Nonetheless, future studies conducted across multiple locations and seasons would help verify the stability of these associations under diverse environmental conditions.

A total of 580 maize inbred lines were used to analyze the genetic structure of quality-related traits in maize stalks. Two replicates were used under a single environmental condition to investigate five quality traits: CP, CF, ADF, NDF, and starch. A wide range of variation was observed, with coefficients of variation ranging from 0.94% to 7.14%. Compared with previous studies that employed association analysis to identify candidate genes related to silage quality [16,17,18,19], this study involved a larger population size and broader variation range. Based on the population structure of the association panel, the lines were divided into two subgroups, and strong correlations among the five quality traits were observed. According to PCA, the phenotypes of these traits were grouped into two categories, with the first two components explaining 73.31% of the total variation. These results indicate that the major quality traits in the population are genetically controlled and suitable for quantitative genetic analysis, which is consistent with earlier studies [20,21,22,23].

### 4.2. Genetic Basis of Quality-Related Traits

Population structure can lead to unpredictable LD patterns across chromosomes, increasing the risk of false positives in genome-wide association studies [24]. In this study, a mixed linear model that incorporated both population structure and genetic relatedness was used to analyze the genetic basis of the five quality traits in silage maize, effectively controlling for false-positive associations.

GWAS has become a widely adopted approach for dissecting the genetic basis of complex quantitative traits in maize. Due to the rapid decay of LD in the maize genome, LD blocks are typically small, allowing high-resolution mapping using dense SNP markers. Here, GWAS using 40,124 high-quality SNPs identified loci significantly associated with CP, CF, ADF, NDF, and starch. When high-density markers are used, LD among background markers can affect multiple testing corrections. The Bonferroni correction is often overly stringent, limiting the ability to detect true associations. To address this, earlier studies have applied alternative thresholds using permutation or sampling-based methods [25,26]. In this study, a significance threshold of *p* < 1.0 × 10^−4^ was adopted, leading to the identification of 18 loci significantly associated with quality traits.

To date, hundreds of QTLs related to nutritional and digestibility traits have been mapped in the maize genome [5,10,27,28]. Prior studies have shown that such traits are often controlled by a few major QTLs and numerous minor ones [10]. Reviews of previous QTL mapping results indicate that QTLs related to digestibility cover 77% and 58% of the maize genome, respectively. The quality-related SNPs identified here were located on eight chromosomes. Due to the reduced detection power of GWAS for rare alleles in maize, no major effect loci were detected in this study. Therefore, combining association analysis with linkage analysis may provide a more comprehensive understanding of the genetic architecture of silage maize quality traits.

### 4.3. Analysis of Genetic Basis of Quality Traits Using GWAS and Meta-Analysis

Since the late 1980s, advances in genetic analysis technologies and quantitative genetic methods have enabled the mapping of numerous QTLs related to maize quality traits [29]. Some loci controlling these traits, including both major and minor genes, have been cloned. To further explore inheritance patterns and molecular mechanisms related to silage maize quality, cQTLs were identified through meta-analysis by integrating QTLs reported in previous studies. A total of 477 QTLs from 47 mapping populations were compiled. These QTLs were not uniformly distributed across chromosomes; chromosome 1 had the most (44), while chromosome 7 had the fewest (21). Independent of genetic background, meta-analysis highlighted that the most limiting factors for stable QTL identification were phenotypic variation and marker density across different environments and years [30,31]. Compared to QTL mapping, association mapping offers a higher resolution for identifying genomic regions related to quantitative traits. However, differences in population structure can introduce false-positive results. Thus, meta-analysis is considered one of the most reliable methods for identifying stable loci. In this study, 26, 8, 18, 13, and 22 cQTLs were identified for CP, CF, NDF, ADF, and starch, respectively. The physical distances of these cQTLs ranged from 2.38 to 86.21 Mb. Seven cQTL intervals showed overlapping signals for multiple traits. Meta-analysis not only improved gene prediction accuracy but also reduced the number of candidate genes within QTL intervals, thereby increasing the efficiency of candidate gene identification [32,33].

While GWAS and meta-analysis reveal the genetic basis of maize quality traits from a genomic perspective, they have inherent limitations [34]. Both methods can be influenced by population structure when identifying regulatory genes, making it difficult to accurately detect small-effect loci or genes. This can lead to both false positives and false negatives. Therefore, integrative analyses involving genomics, transcriptomics, proteomics, and metabolomics are recommended. Multi-omics approaches can complement each method’s limitations and provide a comprehensive understanding of trait genetics [35,36]. In this study, the physical locations of cQTLs were compared with the positions of significant SNPs. Several SNPs were found within cQTL regions. Notably, an AM signal was identified within each cQTL interval for CP, NDF, and starch. This overlap supports the reliability of these AM signals and highlights their potential utility for downstream candidate gene identification [37].

### 4.4. Functional Predictive Analysis of Candidate Genes

The candidate genes identified through the integration of GWAS and meta-analysis were selected based on significant genomic signals and existing annotation databases, offering valuable predictive insights into their potential functional roles. However, given that this study did not include experimental validation, caution should be exercised when interpreting the functional significance of these genes. Further verification using RNA-seq, RT-qPCR, gene editing, or other molecular biology techniques is essential to confirm the predicted functions.

In this study, three candidate genes were identified using both GWAS and meta-analysis, and a total of one, six, and six candidate genes were associated with CP, NDF, and starch, respectively. The functional roles of these genes were examined through GO and KEGG enrichment analyses. The encoded proteins were primarily associated with biological processes such as cellular processes, metabolic processes, biological regulation, stimulus-response signaling, metabolic pathways, biosynthesis of secondary metabolites, ribosomal function, carbon metabolism, protein processing in the endoplasmic reticulum, and amino acid biosynthesis. The gene *Zm00001d037663*, associated with CF, encodes NADH ubiquinone oxidoreductase, an enzyme ubiquitous in eukaryotes and typically localized on the plasma membrane or phagosomes [38,39]. Although limited research exists on NADH oxidoreductase genes (RBOH) in monocotyledonous crops, some studies suggest that *ZmRBOH* may participate in the abscisic acid signaling pathway in maize seedlings and regulate cell wall strength and protoplast capacity [40,41]. To date, there are no published reports on the cloning of this gene in maize. The gene *Zm00001d045078*, associated with ADF, encodes UDP-glucuronic acid 4-epimerase, a key enzyme in pectin polysaccharide biosynthesis. This enzyme is primarily located in the primary cell wall and plays a role in cell adhesion. In *Arabidopsis thaliana*, a pectin biosynthesis mutant known as *emb30* has been characterized, which exhibits defects in cell wall structure, polarity differentiation, and cell division [42,43]. The gene *Zm00001d050977*, associated with starch, belongs to the cytochrome P450 family. *ZmC3H2*, another gene related to cell wall synthesis, is a critical gene in the lignin biosynthesis pathway and encodes a 3-hydroxylase in the CYP98 family. This enzyme catalyzes the hydroxylation of the coumaric acid benzene ring. In the lignin biosynthetic pathway, the C3H gene represents a branching point between H-type and G/S-type lignin monomers [44,45,46]. In *A. thaliana*, a C3H-deficient mutant (*ref*) exhibits significantly reduced lignin content and lower G and S lignin monomer levels [47]. Therefore, cytochrome P450 genes, including *ZmC3H2*, may play key roles in lignin biosynthesis and the regulation of silage quality traits in maize.

Previous studies have identified ten important genes related to silage quality through candidate gene association analysis. Significant associations between polymorphisms in *PAL*, *ZmC3HI*, and *F5H* genes and digestibility-related traits have been reported. However, these associations are often unstable due to population structure and multiple testing. Only a few loci—such as *4CL1*, *CcoAOMT2*, and *ZmPox3*—have demonstrated stable and significant correlations with cell wall digestibility [18,19,48,49,50]. In this study, candidate genes involved in cell wall synthesis metabolism were detected through both GWAS and meta-analysis. Most of the identified genes encode transcription factors or enzymes involved in various biosynthetic pathways. Therefore, we infer that in different association populations, the genetic architecture of silage quality traits is primarily governed by a limited number of key rate-limiting enzyme genes and is further modulated by transcriptional regulatory factors influencing trait phenotypes.

## Figures and Tables

**Table 1:** Descriptive statistics of quality traits in silage maize inbred lines.

Trait	Minimum Value	Maximum Value	Average Value	CV (%)	Skewness	Kurtosis
CP	6.06	6.77	6.57 ± 0.099	1.51	−1.173	2.556
CF	7.47	8.00	7.69 ± 0.092	1.20	−0.017	0.501
NDF	44.05	56.27	49.92 + 2.098	4.20	0.437	0.01
ADF	24.27	45.55	29.05 ± 2.074	7.14	1.573	0.497
Starch	21.97	29.21	24.51 ± 0.906	3.70	0.367	1.865

CP: crude protein, CF: crude fat, NDF: neutral detergent fiber, ADF: acidic detergent fiber.

## Data Availability

The original contributions presented in this study are included in the article/Supplementary Materials. Further inquiries can be directed to the corresponding authors.

## Supplementary Materials

The following supporting information can be downloaded at: https://www.mdpi.com/article/10.3390/plants14152250/s1, Table S1: SNP loci with significant quality-related traits; Table S2: Summary of the collected QTL for quality and quality component traits; Table S3: cQTL Consensus QTL detected by meta-QTL analysis; Table S4: Candidate gene functional annotation.
